# Heavy metals immobilization and bioavailability in multi-metal contaminated soil under ryegrass cultivation as affected by ZnO and MnO_2_ nanoparticle-modified biochar

**DOI:** 10.1038/s41598-024-61270-5

**Published:** 2024-05-09

**Authors:** Mahboobeh Varnaseri Ghandali, Sedigheh Safarzadeh, Reza Ghasemi-Fasaei, Sedigheh Zeinali

**Affiliations:** 1 Department of Soil Science, School of Agriculture, Shiraz University, Shiraz, Iran; 2 Faculty of Advanced Technology, Shiraz University, Shiraz, Iran

**Keywords:** Environmental sciences, Nanoscience and technology

## Abstract

Pollution by heavy metals (HMs) has become a global problem for agriculture and the environment. In this study, the effects of pristine biochar and biochar modified with manganese dioxide (BC@MnO_2_) and zinc oxide (BC@ZnO) nanoparticles on the immobilization and bioavailability of Pb, Cd, Zn, and Ni in soil under ryegrass (*Lolium perenne* L.) cultivation were investigated. The results of SEM–EDX, FTIR, and XRD showed that ZnO and MnO_2_ nanoparticles were successfully loaded onto biochar. The results showed that BC, BC@MnO_2_ and BC@ZnO treatments significantly increased shoots and roots dry weight of ryegrass compared to the control. The maximum dry weight of root and shoot (1.365 g pot^−1^ and 4.163 g pot^−1^, respectively) was reached at 1% BC@MnO_2_. The HMs uptake by ryegrass roots and shoots decreased significantly after addition of amendments. The lowest Pb, Cd, Zn and Ni uptake in the plant shoot (13.176, 24.92, 32.407, and 53.88 µg pot^−1^, respectively) was obtained in the 1% BC@MnO_2_ treatment. Modified biochar was more successful in reducing HMs uptake by ryegrass and improving plant growth than pristine biochar and can therefore be used as an efficient and cost effective amendment for the remediation of HMs contaminated soils. The lowest HMs translocation (TF) and bioconcentration factors were related to the 1% BC@MnO_2_ treatment. Therefore, BC@MnO_2_ was the most successful treatment for HMs immobilization in soil. Also, a comparison of the TF values of plant showed that ryegrass had a good ability to accumulate all studied HMs in its roots, and it is a suitable plant for HMs phytostabilization.

## Introduction

Heavy metal (HMs) pollution caused by industrialization and economic development has become a major environmental, agricultural, and public health problem worldwide^1,2^. Heavy metal are non-degradable and stable in nature, and at concentrations of less than 1 ppm, they are considered toxic to plants and animals^3–6^. Moreover, due to their high mobility, these metals pose a major threat to human and animal health as they accumulate in water bodies and agricultural soils and enter the food chain^7–10^. Therefore, in order to clean and restore soil health and eliminate or limit the bioavailability of HMs to plants and humans, it is essential to use environmentally sound and economically viable technologies^11–13^. To date, various methods have been used to remediate soils contaminated with HMs, including electrokinetic remediation, bioremediation, soil washing, and chemical precipitation^14–17^, but the application of these technologies is limited due to their high cost, environmental contamination from the generation of secondary chemicals, and damage to soil structure^15,16,18,19^. The Environmental Protection Agency (EPA) has identified soil immobilization as the most effective, economical, and environmentally safe technique for remediation of HM-contaminated soils on a broad scale^14,20,21^. Immobilization using ion exchange reactions changes the adsorption, complexation, and precipitation of HMs from active to stable phases and reduces the bioavailability of HMs in soil^22–25^. In situ immobilization of HMs requires careful consideration of the choice of immobilization substance^26^. Many substances such as montmorillonite, limestone, compost, metal oxides, and biochar have been used to immobilize HMs in the soil^7,27,28^.

Biochar is a carbon-rich adsorbent produced from the pyrolysis of various raw materials in the absence or limited presence of oxygen^29–31^. Due to its high porosity, active functional groups, and high CEC, biochar has been considered an ideal amendment for the immobilization of HMs in^24,32,33^. Therefore, biochar can reduce the availability, mobility and leaching of HMs in soil, and limiting their bioavailability for the plants^15,30^. Biochar immobilizes HMs in soil through various mechanisms such as complexation, cation exchange, electrostatic interactions, reduction and precipitation^30^. Biochar also protects the plant from HMs by increasing antioxidant activity^31^. However, unmodified biochar has a limited capacity to immobilize and adsorb HM in soil^23,24,34,35^. Therefore, it is important to modify biochar to improve its ability to immobilize HMs in soil. Various physical, chemical, and biological techniques have been used to improve the properties of biochar, e.g. specific surface area, porosity, number of adsorption sites on the surface, and number of surface functional groups^36–38^. Recently, the modification of biochar with nanomaterials, especially nanometals, has attracted attention due to their high efficiency, low toxicity and environmental compatibility^39–41^. Nanomaterials are particles ranging in size from 1 to 100 nm^42,43^. In the last decade, nanomaterials have been used extensively for the removal of HMs from water and soil due to their tiny size, strong reactivity, and high surface activity^44–46^. However, these materials tend to aggregate and deactivate due to their high surface energy, which limits their ability to remove contaminants^33,41,44–47^. To overcome these problems, biochar can serve as a carrier for metal oxide nanoparticles due to its porosity and high ion exchange capacity^48,49^. The composite of biochar and metal nanoparticles eliminates their deficiencies and creates a material that combines the advantages of both properties^50,51^. The functional surface groups, the specific surface area, and the cation exchange capacity increase by modifying biochar with metal nanoparticles. This increase leads to improvement of ion exchange, complexation and thus the stabilization of HMs^36,49,52^. Among metal nanoparticles, zinc oxide nanoparticles have attracted attention due to their low toxicity, compatibility, high binding energy, low production cost and biodegradability compared to other nanoparticles^44,53,54^. They are also widely used for the removal of HMs from water and soil^39^. In addition, manganese oxide nanoparticles have been shown to be well suited for stabilization of HMs due to their high specific surface area, high affinity for HMs, abundance of hydroxyl functional groups on their surface, stability in a wide pH range, and high negative surface charges^55–58^.

Numerous studies have demonstrated the effect of biochar modified with various nanoparticles on the immobilization of HMs in soil^37,59–62^. However, studies on the effect of biochar modified with ZnO and MnO_2_ nanoparticles on HMs immobilization in soil under plant cultivation is still limited and need to be explored in detail. Ryegrass (*Lolium perenne* L.) was selected as the study plant in our research due to its fast growth, high biomass, high potential for accumulation of metals, and high tolerance to these metals^63–65^. In this study, we tested the hypothesis that MnO_2_ and ZnO nanoparticle-modified biochars outperforms the pristine biochar in terms of their ability to immobilize HMs, reduce their bioavailability, and improve plant development. Therefore, the objectives of this study include: (i) Synthesis of modified biochar with MnO_2_ and ZnO nanoparticles and comparison their morphology, physicochemical properties (ii) Evaluation and comparison of the efficiency of pristine biochar (BC), biochar modified with ZnO nanoparticles (BC@ZnO), and biochar modified with MnO_2_ nanoparticles (BC@MnO_2_) in term of immobilization of Pb, Cd, Ni and Zn in multi-metal contaminated soils and (iii) Comparing the effects of pristine and ZnO and MnO_2_ nanoparticle modified biochar on the HMs uptake and ryegrass (*Lolium perenne* L.) growth.

## Results

### Characteristics of the BC, BC@MnO_2_ and BC@ZnO

#### FT-IR analysis

**Figure 1:**
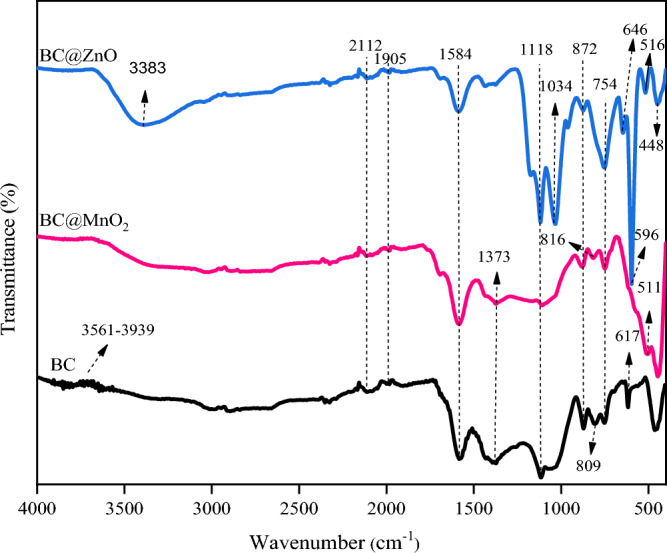
FTIR spectra of BC, BC@MnO_2_ and BC@ZnO.

FTIR analysis was carried out to investigate the functional groups of BC, BC@MnO_2_, and BC@ZnO (Fig. 1). The broad band 3300–3940 cm^−1^ represents the strong and broad O–H stretching of alkyne^66,67^. The peaks at 2700–3000 cm^−1^ corresponded to the C–H stretching vibration^68,69^. The existence of the C ≡ C triple or C = C = C double bonds of alkene was confirmed by the peaks at 1905 and 2112 cm^−1^^70^. The peak at 1584 cm^−1^ is attributed to the stretching vibration of C = C^71^. The peak at 1373 cm^−1^ was the characteristic peak of –C–H of the methyl group^72^. The peaks at 1116 cm^−1^ are assigned to C–O–C stretching vibrations in ether or saturated chain anhydrides^73,74^. The peak at 1034 cm^−1^ is assigned to C–O stretching^75^. The peaks at 729–880 cm^−1^ were characteristic of the bending vibration of aromatic C–H groups^76^. The peak at 617 cm^−1^ is attributed to the bending vibration of the O–H group^77^. The peak at 511 cm^−1^ is associated with the Mn–O stretching vibration, shows that the MnO_2_ nanoparticles were successfully loaded onto the surface of the biochar^66,73^. Peaks at 646, 596, 516 and 448 cm^−1^ confirmed the presence of a Zn–O stretching vibration^77–79^.

#### XRD analysis

**Figure 2:**
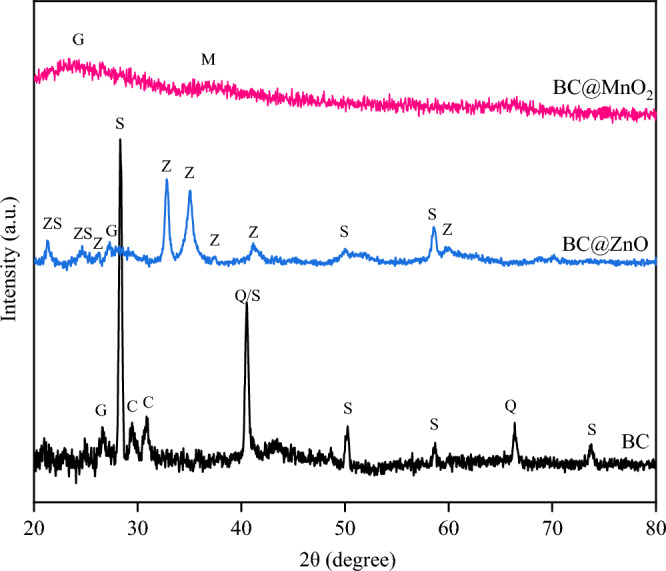
XRD patterns of BC, BC@ZnO and BC@MnO_2_ (G: graphite_,_ M: MnO_2_, ZS: zinc silicate, Z: wurtzite ZnO, S: sylvite, Q: quartz C: calcite).

To determine the crystal structure and phase composition of BC, BC@MnO_2_, and BC@ZnO, the X-ray diffraction (XRD) pattern was used (Fig. 2). The peaks of 26.6° in BC, 27.3° in BC@ZnO and 23° in BC@MnO_2_ were attributed to graphite, indicating that a stable and regular graphite structure was formed during the pyrolysis process^80^. The peak at 40.6° in BC was assigned to quartz or sylvite^81,82^. The peaks at 50.2° and 58.72° in BC and BC@ZnO and the peaks at 28.4° and 73.7° in BC, corresponded to sylvite^83–85^. The peaks at 29.4° and 30.9° in BC were attributed to calcite^74^. The peak at 66.4° in BC was assigned to quartz^74^. The peaks at 26.3°, 32.93°, 34.8°, 37.5°, 41.2° and 60.2° reflect the crystal planes of the hexagonal structure of wurtzite ZnO recorded in the card (JCPDS card No. 36–1451)^78,86–88^. The 21.4° and 24.5° peaks in BC@ZnO are also indicative of zinc silicate^78,86–89^. The XRD pattern of BC@ZnO is consistent with the results of other related studies^90–93^. The 36.7° peak in BC@MnO_2_ was assigned to amorphous MnO_2_^73,94^. Compared to the patterns of BC and BC@ZnO, the XRD pattern of BC@MnO_2_ revealed weaker peaks. These weak peaks were probably caused by the charge of the amorphous MnO_2_, which had an impact on the XRD patterns of other crystals^66^. In general, the XRD of MnO_2_-loaded biochar did not show many peaks for phase identification. This could be due to the unfavorable effect of the high reaction rate between Mn(II) and Mn(VII), in the case of MnO_2_ synthesized by the co-precipitation method^73,95^. According to the results of previous studies, amorphous materials have effective adsorption properties due to their high specific surface area and the large number of active sites on their surfaces^73,96^. These results are consistent with the observations of this study, which showed that BC@MnO_2_ plays a more effective role in the surface adsorption of heavy metals compared to other adsorbents.

#### SEM–EDX and BET analysis

**Figure 3:**
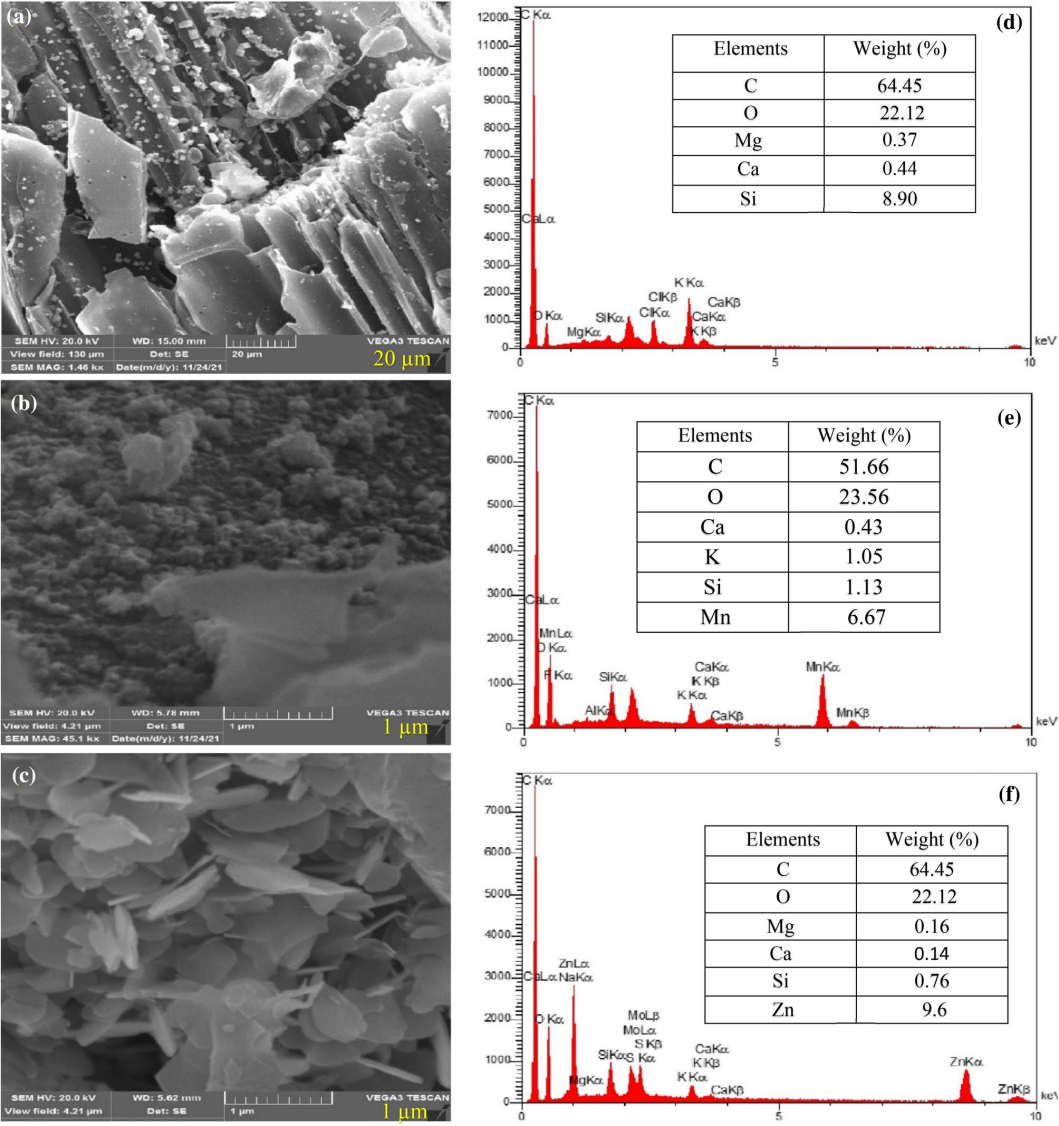
SEM images of BC (**a**), BC@MnO_2_ (**b**) and BC@ZnO (**c**) and EDX spectrum of BC (**d**), BC@MnO_2_ (**e**) and BC@ZnO (**f**).

The surface morphology and elemental composition of BC, BC@MnO_2_ and BC@ZnO were investigated using SEM–EDX analysis (Fig. 3). According to Fig. 3a, the biochar contains honeycomb and porous structures, which are probably due to the removal of volatile substances during the pyrolysis process. Figure 3b shows that the surface of biochar is uniformly covered with fine and dense particles, indicating the deposition of MnO_2_ nanoparticles. Similar results were obtained by^20,66,97^. The SEM of the BC@ZnO nanocomposite showed spherical and white particulates of ZnO in the form of clusters on the biochar surface. These spherical particles are probably due to the irregular growth of the ZnO crystals^98^. These results have also been obtained by other researchers^88,93,99–101^. In general, the surface of the biochar modified with ZnO and MnO_2_ nanoparticles was irregular, non-uniform and more uneven compared to pristine biochar confirming the successful loading of ZnO and MnO_2_ nanoparticles onto the biochar. Previous research has shown that a rough surface leads to an increase in the specific surface area and increase in the positive charge thereby increasing the adsorption of pollutant^20,99,100,102,103^.

EDX analysis of the biochar showed the presence of C, O, Ca, Mg, K, Si and Cl elements. The presence of Mn and Zn and the significant increase in O content in BC@MnO_2_ and BC@ZnO confirmed the successful loading of the biochar surface with MnO_2_ and ZnO nanoparticles.

Therefore, according to the results of XRD, FTIR and SEM–EDX analyses, it is likely that the ZnO and MnO_2_ nanoparticles were successfully loaded onto the BC.

The BET analysis showed that the addition of MnO_2_ and ZnO nanoparticles increased the specific surface area of pristine biochar from 60.52 m^2^ g^-1^ to 430.71 and 341.2 m^2^ g^-1^, respectively. This increase is most likely the result of the deposition of ZnO and MnO_2_ nanoparticles on the surface of biochar, which led to an increase in surface roughness and consequently an increase in specific surface area^82,104^. It can be concluded that biochar modified with nanoparticles has appropriate active sites for the adsorption of HMs, making it a high-potential adsorbent. In similar studies, Liang et al. (2017) and Wang et al. (2020), reported that loading biochar with MnO_2_ and ZnO nanoparticles led to a significant increase in specific surface area of nanocomposites^66,88^. According to the results, biochar modified with MnO_2_ nanoparticles can absorb heavy metals more effectively compared to other adsorbents. This superiority can be attributed to the amorphous structure, more exchangeable cations and a higher specific surface area^133^.

### Effect of the adsorbents on ryegrass growth

**Figure 4:**
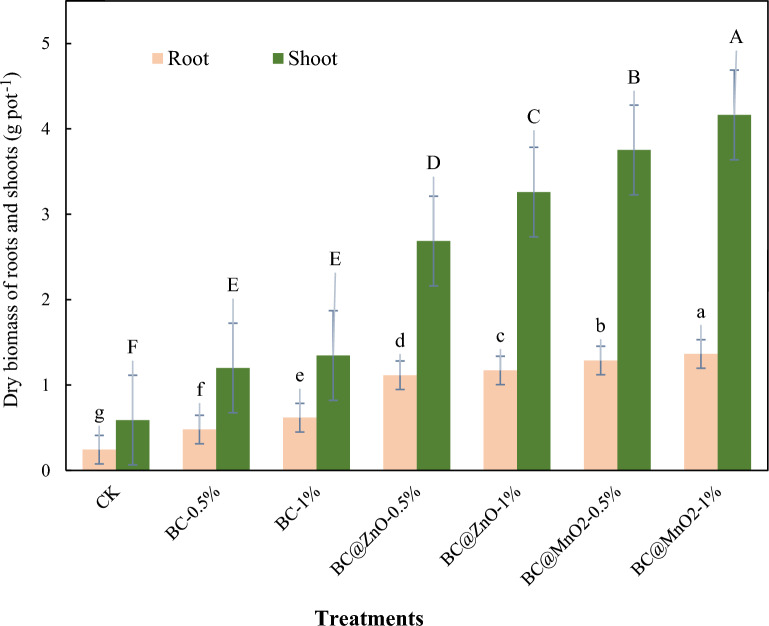
Effect of BC, BC@ZnO and BC@MnO_2_ application on dry weight of ryegrass root and shoots. HMs polluted soil (Control) (CK), pristine biochar (0.5% (BC-0.5%), 1% (BC-1%)), ZnO NPs-modified biochar (0.5% (BC@ZnO-0.5%), 1% (BC@ZnO-1%)) and MnO_2_ NPs-modified biochar (0.5% (BC@MnO_2_-0.5%), 1% (BC@MnO_2_-1%)). Different letters over the bars indicate a significant difference between treatments according to Duncan’s test (*P* < 0.05).

To investigate the effects of BC, BC@ZnO, and BC@MnO_2_ at doses of 0.5 and 1% on the growth of ryegrass under Cd, Pb, Zn, and Ni stress, the dry weight of the roots and shoots of ryegrass was evaluated (Fig. 4). Compared to the controls, the application of BC, BC@MnO_2_, and ZnO significantly (*p* < 0.05) increased the dry weight of the ryegrass shoots and roots. The application of biochar at 0.5% and 1% increased the dry weight of ryegrass shoots and roots by about 50.83–61% and 60.56–49.17%, respectively, compared to the control treatment. The addition of BC@ZnO and BC@MnO_2_ at 0.5% and 1% also increased the dry weight of the aerial parts by 78–81.9% and 84.28–85.83%, respectively, compared to the control. The addition of BC@MnO_2_ and BC@ZnO at 0.5% and 1% increased the root dry weight by 78.11–79.2% and 81–82.13%, respectively, compared to the control treatment. The dry weight of ryegrass roots and shoots in biochar treatments modified with ZnO and MnO_2_ nanoparticles differed significantly (*p* < 0.05) compared to the pristine biochar treatment. Moreover, the addition of BC@MnO_2_ to the soil significantly increased the dry weight of the root and shoot biomass of ryegrass compared to the BC@ZnO treatments (*p* < 0.05). The type and dose of adsorbent also had a significant effect on the dry weight of roots and shoots. Increasing the dose from 0.5 to 1% significantly increased the dry weight of the roots and shoots. The results from Fig. 4 show that the BC@MnO_2_ treatment resulted in the highest dry weight of roots and shoots, while the control treatment gave the lowest. BC@MnO_2_ was found to be more effective than BC@ZnO in increasing the dry weight of roots and shoots. In addition, a positive correlation was found between the shoot and root dry weights of the ryegrass plants in all treatments.

After increasing the adsorbent dosage from 0.5 to 1%, the dry weight of the roots and shoots increased. This increase is probably the result of increased HMs immobilization in the soil and improved nutrient supply at higher doses. In agreement with previous studies, a linear relationship was found between the amount of adsorbent used and the dry weight of the root and shoot of plant^11,105,106^.

### Effects of BC, BC@MnO_2_, and BC@ZnO on the uptake of Ni, Zn, Pb, and Cd by ryegrass shoots and roots

Table 1 shows data on HM uptake by ryegrass shoots treated with BC, BC@MnO_2_, and BC@ZnO. Compared to the control treatment, the addition of pristine biochar and biochar modified with nanoparticles significantly reduced (*p* < 0.05) the uptake of Pb, Ni, and Cd in the ryegrass shoot. The application of 0.5% and 1% BC, BC@ZnO, and BC@MnO_2_ reduced Pb uptake in the shoots by 22.29–47.31%, 67.55–74.79%, and 83.82–89.33%, respectively, compared to the control. Application of BC, BC@ZnO, and BC@MnO_2_ at two levels of 0.5% and 1% reduced uptake of Cd in shoot parts by 18.97–45.11%, 75.29–78.37%, and 78.73–82.83%, respectively, compared to control. Table 1 also shows that the application of 0.5 and 1% BC, BC@ZnO and BC@MnO_2_ decreased the Ni uptake in the shoots by 11.55–32.58%, 52.76–61.67%, and 68.44–71.88%, respectively, compared to the control. Although Zn uptake in shoots was significantly (*p* < 0.05) decreased by different doses of BC (17.86–40.20%) and BC@MnO_2_ (74.98–79.85%) compared to control, BC@ZnO significantly (*p* < 0.05) increased Zn uptake in shoots (4.91–16.61%). Compared to application of BC treatments, the application of BC@MnO_2_ and BC@ZnO treatments resulted in a greater reduction in the uptake of HMs in the shoots, with the exception of ZnO nanoparticle-modified biochar, which increased the uptake of Zn in the shoots. In addition, BC@MnO_2_ was more effective than BC@ZnO in reducing HMs uptake by the shoots. In addition to the type of adsorbent, the amount of adsorbent also affected the uptake of HMs by the shoots of ryegrass. When the rates of BC, BC@ZnO, and BC@MnO_2_ increased from 0.5 to 1%, the Pb, Ni and Cd uptake in shoots decreased. Zn uptake in shoots increased by increasing the amount of BC@ZnO from 0.5 to 1%. Also, BC@MnO_2_ was more effective than BC@ZnO in decreasing the uptake of HMs by the shoots. According to our results, the lowest uptake of HMs by ryegrass shoots was observed in 1% of BC@MnO_2_ treatment. Moreoveer, all adsorbents showed a strong propensity to uptake Pb than other HMs.

**Table 1:** Effect of BC, BC@ZnO and BC@MnO_2_ on the uptake of HMs by ryegrass shoots (μg pot^-1^).

Treatments	Pb	Cd	Zn	Ni
CK	123.54 ± 0.12^a^	145.17 ± 0.46^a^	168.79 ± 0.98^c^	191.63 ± 0.36^a^
BC-0.5%	96.01 ± 0.47^b^	117.63 ± 0.67^b^	132.08 ± 0.14^a^	169.49 ± 0.49^b^
BC-1%	65.09 ± 0.36^c^	79.68 ± 0.87^c^	96.15 ± 0.15^e^	129.19 ± 0.89^c^
BC@ZnO-0.5%	40.08 ± 0.19^d^	35.87 ± 0.48^d^	168.69 ± 0.74^b^	90.52 ± 0.63^d^
BC@ZnO-1%	31.15 ± 0.16^e^	31.40 ± 0.16^e^	187.50 ± 0.29^b^	73.45 ± 0.79^e^
BC@MnO_2_-0.5%	19.99 ± 0.5f.	30.88 ± 0.28^e^	40.24 ± 0.18f.	60.48 ± 0.48f.
BC@MnO_2_-1%	13.18 ± 0.11^g^	24.92 ± 0.1f.	32.41 ± 0.22^g^	53.88 ± 0.13^g^

HMs polluted soil (Control) (CK), pristine biochar (0.5% (BC-0.5%), 1% (BC-1%)), ZnO NPs-modified biochar (0.5% (BC@ZnO-0.5%), 1% (BC@ZnO-1%)) and MnO_2_ NPs-modified biochar (0.5% (BC@MnO_2_-0.5%), 1% (BC@MnO_2_-1%)). Values are expressed as mean ± standard error (*n* = 3). Values with different letters indicate a significant difference between treatments according to Duncan’s test (*P* < 0.05).

Table 2 shows the effect of BC, BC@MnO_2_, and BC@ZnO at 0.5 and 1% on the uptake of HMs by ryegrass roots. Compared to the control treatment, the addition of BC, BC@MnO_2_, and BC@ZnO significantly reduced the uptake of Ni, Pb, and Cd by plant roots (*p* < 0.05). Compared to the control, Zn uptake was significantly reduced (*p* < 0.05) by the BC and BC@MnO_2_ treatments but increased by BC@ZnO. With the exception of the BC@ZnO treatment, which increased the Zn uptake, the application of biochar modified with nanoparticles decreased HMs uptake in roots more than pristine biochar. Moreover, the dosage of the adsorbents proved to be influential in reducing the HMs uptake by the ryegrass roots. When the amount of BC was increased from 0.5 to 1%, uptake of Pb, Cd, Zn, and Ni in root decreased by 16.74, 16.74, 15.71, and 22.19%, respectively. Similarly, increasing the BC@MnO_2_ dosage from 0.5 to 1% decreased the root uptake of Cd, Pb, Zn, and Ni by 16.46%, 25.2%, 20.2%, and 11.18%, respectively. . The absorption of Cd, Ni, and Pb in the roots decreased by 16.27%, 19.7%, and 16.15%, respectively, when in the BC@ZnO dose was increased, while Zn uptake increased by 12.79%. As the results show, the roots of ryegrass had higher concentrations of HMs compared to the shoots.

**Table 2:** Effect of BC, BC@ZnO and BC@MnO_2_ on the HMs uptake by ryegrass roots (μg pot^-1^).

Treatments	Pb	Cd	Zn	Ni
CK	190.22 ± 0.69^a^	221.84 ± 1.11^a^	229.58 ± 0.78c	240.79 ± 0.63^a^
BC-0.5%	168.43 ± 0.83^b^	185.58 ± 1.12^b^	197.55 ± 0.47d	226.09 ± 0.76^b^
BC-1%	140.24 ± 0.21^c^	154.52 ± 0.77c	166.52 ± 0.57^e^	161.14 ± 0.37^c^
BC@ZnO-0.5%	111.16 ± 0.32^d^	80.43 ± 0.65^d^	303.74 ± 0.53^b^	124.87 ± 0.72^d^
BC@ZnO-1%	89.26 ± 0.55^e^	67.35 ± 0.44^e^	342.58 ± 0.41^a^	104.70 ± 0.3^e^
BC@MnO_2_-0.5%	56.50 ± 0.35f.	79.47 ± 0.15^d^	93.29 ± 0.4f.	94.50 ± 0.41f.
BC@MnO_2_-1%	42.26 ± 0.37^ g^	66.39 ± 0.62^e^	74.48 ± 0.2^ g^	83.93 ± 0.04^ g^

HMs polluted soil (Control) (CK), pristine biochar (0.5% (BC-0.5%), 1% (BC-1%)), ZnO NPs-modified biochar (0.5% (BC@ZnO-0.5%), 1% (BC@ZnO-1%)) and MnO_2_ NPs-modified biochar (0.5% (BC@MnO_2_-0.5%), 1% (BC@MnO_2_-1%)). Values are expressed as mean ± standard error (*n* = 3). Values with different letters indicate a significant difference between treatments according to Duncan’s test (*P* < 0.05).

In general, the tendency for uptake HMs by BC and BC@MnO_2_ was as Pb > Cd > Zn > Ni and for BC@ZnO as Pb > Cd > Ni > Zn in the roots and shoots of ryegrass; in reality, the plant absorbs these heavy metals to a lesser extent. The lower uptake of heavy metals by the plant leads to a higher immobilization of heavy metals in the soil, and thus to lower availability for the plant.

### Bio-concentration (BCF) and translocation factors (TF)

**Figure 5:**
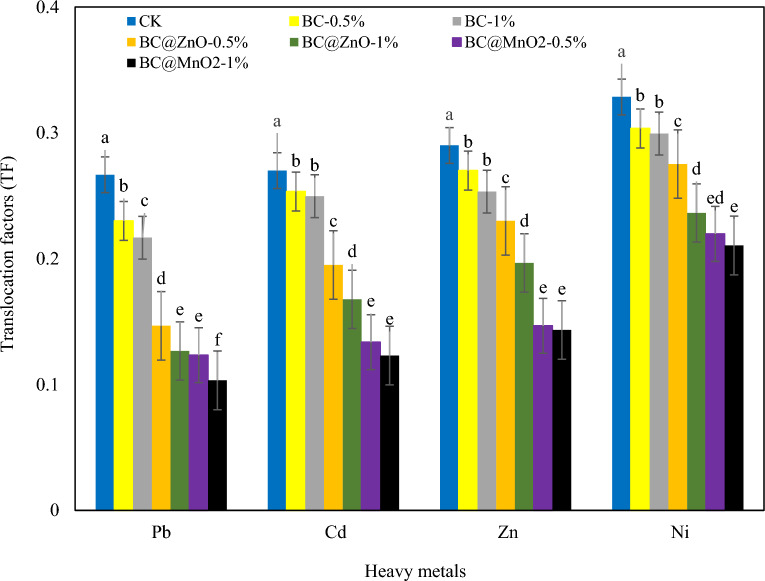
Influence of BC, BC@ZnO and BC@MnO_2_ on translocation factors (TF). HMs polluted soil (Control) (CK), pristine biochar (0.5% (BC-0.5%), 1% (BC-1%)), ZnO NPs-modified biochar (0.5% (BC@ZnO-0.5%), 1% (BC@ZnO-1%)) and MnO_2_ NPs-modified biochar (0.5% (BC@MnO_2_-0.5%), 1% (BC@MnO_2_-1%)). Different letters over the bars indicate a significant difference according to Duncan’s test (*P* < 0.05).

**Figure 6:**
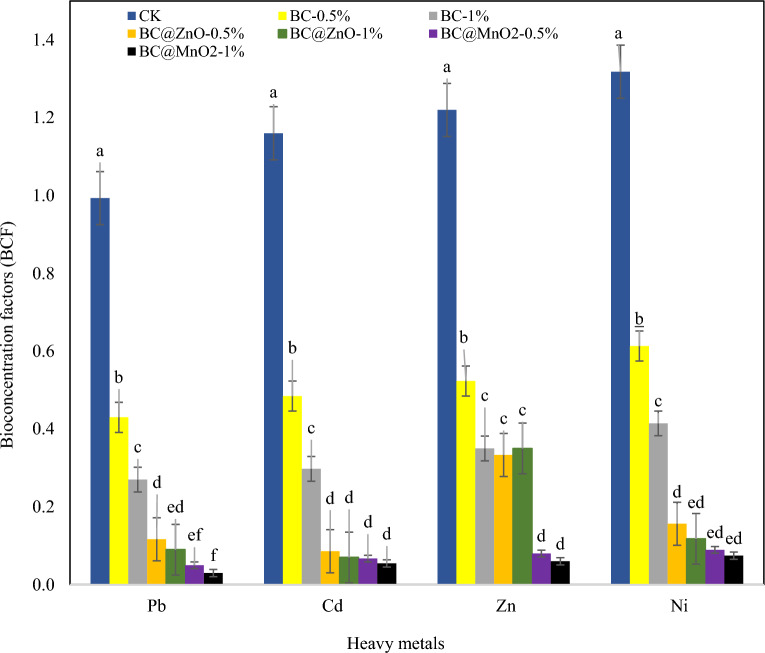
Influence of BC, BC@ZnO and BC@MnO_2_ on bio-concentration factors (BCF). HMs polluted soil (Control) (CK), pristine biochar (0.5% (BC-0.5%), 1% (BC-1%)), ZnO NPs-modified biochar (0.5% (BC@ZnO-0.5%), 1% (BC@ZnO-1%)) and MnO_2_ NPs-modified biochar (0.5% (BC@MnO_2_-0.5%), 1% (BC@MnO_2_-1%)). Different letters over the bars indicate a significant difference according to Duncan’s test (*P* < 0.05).

To better understand the effect of different amendments on the uptake and translocation of heavy metals in ryegrass, the bioaccumulation factor (BCF) and translocation factor (TF) were calculated and presented in Figs. 5 and 6. According to the results shown in Fig. 5, the addition of all adsorbents to the soil, significantly decreased (*p* < 0.05) the TF for all HMs compared to the control treatment. Therefore, addition of 0.5% and 1% biochar led to reductions in TF values for Pb, Cd, Zn, and Ni by 13.75–18.75%, 6.17–7.53%, 6.90–12.64%, and 7.63–8.87%, respectively, compared to the control. The effect of BC treatment in reducing the TF of HMs compared to the control was as follows: Pb > Zn > Ni > Cd. Also, addition of BC@ZnO at two levels of 0.5% and 1% decreased the TF values for Pb, Cd, Zn and Ni compared to the control treatment from 45 to 52.50%, 27.78–37.90%, 20.69–32.18%, and 16.23–28.06% respectively,. Compared to the control, BC@ZnO treatment reduced the TF of HMs in the following order: Pb > Cd > Zn > Ni. Similarly, the addition of BC@MnO_2_ at 0.5 and 1% decreased the TF values for Pb, Cd, Zn, and Ni from 53.75 to 61.25%, 50.49–54.44%, 49.43–50.57%, and 33.13–35.93%, respectively, compared to the control treatment. The effect of biochar treatment on reduction of TF values compared to the control were as follows: Pb > Cd > Zn > Ni. Increasing the amount of adsorbent led to a further reduction in TF. As can be seen in Fig. 5, the TF values of HMs in the nanoparticle-modified biochar treatments were significantly lower (*p* < 0.05) than those in the pristine biochar treatment. In addition, BC@MnO_2_ reduced the TF of HMs in ryegrass better than BC@ZnO. The treatment with 1% BC@MnO_2_ had the lowest TF of HMs, while the control treatment had the highest TF. In general, according to the obtained results, the TF of HMs in ryegrass was as follows: Ni > Zn > Cd > Pb, indicating greater transfer of Ni from the roots to the shoots.

Similar to TF, the addition of adsorbent reduced the BCF of HMs compared to the control, and this decrease became greater when the adsorbent dose was increased. According to the results, the control treatment and the treatment with 1% BC@MnO_2_ had the highest and lowest BCF values for HMs, respectively. Thus, the BCF values of Pb, Cd, Zn, and Ni decreased by 96.98, 95.29, 95.08, and 94.35%, respectively in the treatment of 1% BC@MnO_2_ compared to the control. The BCF in the biochar modified with nanoparticles was significantly lower compared to the treatment of pristine BC. In general, the BCF in ryegrass in all treatments was as follows: Ni > Zn > Cd > Pb.

## Discussion

### Effect of BC, BC@MnO_2_ and BC@ZnO on plant biomass

Abiotic stress, such as heavy metals, can lead to physiological changes in plants, including a decrease in plant biomass^107–109^. The lower dry weight of roots and shoots observed in the control treatment compared to the other treatments may be attributed to the high concentration of HMs in the plants (Fig. 4). This results is consistent with previous studies^108,109^. Shoot and root dry weight increased significantly in comparison to the control treatment when biochar and modified biochar were added to metal-contaminated soils. This might be due to the fact that the addition of biochar and modified biochar stabilize of HMs in the soil and limit their bioavailability to the plants, and reduce the uptake of metals leading to an improvement in root and shoot growth of the plant. Similar results were obtained by Ali et al. (2019)^110^ and Shahbaz et al. (2018)^111^. They reported that the application of biochar prevents the transfer of HMs in the plant and increases the dry weight and grain yield of wheat, sunflower, and maize. In addition, biochar increases plant growth by providing macro and micronutrients. In addition, biochar improves the physicochemical properties of the soil, which produces the optimal conditions for better plant growth. These conclusions have been supported by numerous reports.^112–115^. Research by Wang et al. (2021) also aligns with these results and shows that the presence of various biochars improves nutrient availability^116^ and HMs immobilization^117^, resulting in increased ryegrass biomass^18^. The results of the present study have obviously shown that loading of biochar with ZnO and MnO_2_ nanoparticles plays a role in increasing the dry weight of ryegrass roots and shoots (Fig. 4). The increase in the dry weight of roots and shoots in modified biochar treatments may be due to the dual effect of biochar and nanoparticles on the plants. Indeed, the application of biochar immobilizes the HMs in the soil and nanoparticles increase the zinc and manganese content in the plant. These results are in line with published studies showing that NPs promoted the growth of the plants when exposed to metal stress^53,118,119^. These results are consistent with other findings^108,109^. In one study, Kareen et al. (2023) observed that incorporation of biochar with zinc oxide nanoparticles resulted in improved alfalfa growth in Cd-contaminated soil. They attributed this result to the competition between nutrients and heavy metals at the root surface and to the immobilization of Cd in the soil^109^. Lin et al. (2017) and Yu et al. (2017) reported that the application of MnO_2_ modified biochar in an arsenic-contaminated soil significantly increased the grain weight and dry weight of rice roots and shoots compared to pristine biochar^120,121^. In addition, the BC@MnO_2_ treatment performed better compared to other treatments in terms of increasing the dry biomass of ryegrass roots and shoots. This is most likely due to the fact that BC@MnO_2_, due to its higher specific surface area, amorphous structure, and greater number of exchangeable cations, reduced the bioavailability of HMs in the soil more effectively than other treatments thus minimizing their impact on ryegrass development.

### Effect of BC, BC@MnO_2_ and BC@ZnO on plant HMs concentration

Various organic treatments have been employed to reduce the bioavailability and toxicity of HMs in plants and soils,^122,123^. As reported by Azim-Zadeh et al. (2014), organic amendments can alter soil properties, ultimately affecting the bioavailability of HMs^124^. In this study, the presence of HMs in ryegrass shoots and roots was used as an indicator of the HMs bioavailability in soils (Tables 1 and 2). In the present study, addition of BC, BC@MnO_2_ and BC@ZnO significantly decreased the uptake of HMs in the roots and shoots and as the dose was increased, the reduction effect increased. This might be due to the fact that biochar immobilizes HMs in soil and reduces their bioavailability. Previous research has shown that oxygen-containing functional groups on biochar surfaces facilitate the immobilization of metal ions through the formation of precipitates and complexes^27,125,126^. The interaction with the mineral and crystal lattices on the BC surface also allows the HM ions to form to form insoluble inorganic compounds (such as metal phosphate, metal carbonate, or metal silicate)^127,128^. EDX analysis revealed (Fig. 3) that the presence of divalent cations such as Ca (II) and Mg (II) in biochar can replace HM ions on biochar surfaces^129^. In fact, it can be argued that biochar decreased the bioavailability of HMs by immobilizing HMs in the soil through the mechanisms of ion exchange, complexation, and precipitation, which in turn reduced uptake by ryegrass roots and shoots. The silica in wheat straw biochar can also lead to a reduction in bioavailability in the soil, a reduction in adsorption by the plant, and a reduction in the transfer of HMs from the roots to the shoots^130^. Similar results have also been reported by other researchers. Based on the research of Wang et al. (2018), biochar significantly reduced the solubility of HMs in soil^131^. Awad et al. (2020) reported that the HM contents of soil and plant tissues were significantly reduced after biochar applications^132^. The results showed that loading MnO_2_ and ZnO nanoparticles on the biochar led to a more noticeable decrease in the plant's absorption of HMs, suggesting that BC@ZnO and BC@MnO_2_ have a greater ability to immobilize HMs than pristine biochar. BC@NP composites have better physicochemical properties compared to pristine biochar due to the combination of both constituent particles. Therefore, the substantial reduction in HMs uptake by ryegrass with nanoparticle-modified biochar application may be attributed to the combined effects of biochar and nanoparticles on soil and plant surfaces. In addition to the benefits of biochar in reducing the bioavailability of HMs, nanoparticles can also reduce the adverse effects of HMs on plant growth and productivity^27^. Also, according to the results of BET analysis, biochar modified with nanoparticles had a higher specific surface area compared to pristine biochar. This suggests that these treatments have been more effective in immobilizing HMs^133^. According to previous research, cation channels of Ca^2+^, Zn^2+^, Mn^2+^, and Fe^2+^ are the pathways through which Cd, Pb, Ni, and other divalent heavy metals enter plant cells^134,135^. When BC@MnO_2_ and BC@ZnO are applied, Mn^2+^ and Zn^2+^ levels increase, which can compete with HMs in the transport pathway and obstruct their transfer through plant membranes. In similar reports,^2,136,137^ the addition of MnSO_4_ and ZnSO_4_ amendments could greatly reduce the buildup of HMs by increasing the amount of accessible Mn and Zn content. In the study of Wang et al. (2019) the capacities of biochar derived from tea branches and Fe–Mn-modified biochar (MnFe_2_O_4_-biochar) to immobilize Sb and Cd in contaminated soils and reduce the bioavailability of Sb and Cd in *Lolium multiflorum* Lam were assessed. Their results demonstrated that MnFe_2_O_4_-biochar application significantly reduced extractable Sb and Cd concentrations, transformed exchangeable Sb and Cd into less accessible forms, and decreased the accumulation of Sb and Cd in plants^138^. Furthermore, Suleiman et al. (2020) investigated the biochar with ZnO nanoparticles treatment on sunflowers grown in wastewater contaminated with HMs. They reported that biochar and ZnO significantly reduced the availability of HMs and decreased their uptake by sunflower compared to pristine biochar^27^.

As shown in Tables 1 and 2, the BC@ZnO treatment increased Zn^2+^ uptake by the ryegrass roots and shoots. This is probably due to dissolving of the ZnO nanoparticles in soil and releasing ionic Zn, and increasing its bioavailability and uptake by plants. Similar results were obtained by Hossein et al. (2018), Shafqat Ali et al. (2019), and Rizwan et al. (2019). They reported that ZnO nanoparticles increased the bioavailability of Zn^2+^ and increased its uptake by different plant organs^118,139,140^.

The data in Tables 1 and 2 show that there was a significant difference between BC@ZnO and BC@MnO_2_ treatments in reducing the uptake of Pb, Ni, and Zn for the roots and shoots of plants, but no significant difference was observed for Cd.

Previous research has demonstrated that ZnO nanoparticles decrease Cd levels in a variety of plant species^44,141,142^. Due to the antagonistic effects of both metals, it is possible to link this decrease in Cd uptake, at least in part, to the rise in Zn levels^143^. Zn and Cd compete with each other because both are transported to the root surface plasma membrane by a common carrier^144^. Since the adsorption of Zn is easier than that of Cd, Cd phytoaccumulation is reduced when Zn is present^145^. Numerous studies have shown that plants with higher Zn levels absorbed less Cd^22,141,146,147^.

According to our results, the amount of Pb uptake by the plant was lower than that of other metals. This demonstrates that Pb is more adsorbed and immobilized onto the adsorption sites than Cd, Ni, and Zn. Zn, Pb, Cd, and Ni are cations with similar valency, therefore, they are able to compete for the same sites and functional groups on the adsorbent’s surfaces. According to Houben et al. (2013), Pb may have a stronger affinity for carboxylic and phenolic functional groups that are present on the surface of BC^148^. Similar findings were observed by Jiang et al. (2020), Norini et al. (2019), and Namgay et al. (2010), who showed that biochar immobilizes Pb more strongly than other cationic HMs and removes it from the reach of plants^149–151^.

Therefore, according to the results of our study, it can be stated that BC, BC@MnO_2_, and BC@ZnO can effectively decrease the accumulation of HMs in ryegrass, consequently reducing phytotoxicity. Also, biochar modified with nanoparticles were more effective than pristine biochar in reducing the uptake of HMs by ryegrass.

### Effect of BC, BC@MnO_2_ and BC@ZnO on bio-concentration (BCF) and translocation factors (TF) of HM in ryegrass tissues

The two most important metrics for assessing the potential risks of metal ions for plant growth in contaminated soils are bioconcentration (BCF) and translocation factors (TF). TF indicates the transport of HMs from the roots to the shoots of ryegrass. The TF value (Fig. 5) of all HMs was less than 1, indicating that the HMs mainly accumulate in the roots. A low TF value also indicates a lower transport of HMs from roots to the shoots. Thus, all adsorbents were successful in preventing the HMs transport from the roots to the shoots. Compared to pristine biochar, nanoparticle-modified biochar was more effective in reducing the transfer of HMs. The results presented in Fig. 6 indicated that the BCF values of all HMs except Pb (in the control treatment) were greater than 1 and with the addition of adsorbents reached below 1. Therefore, the results confirm that in this study, soil amendment with BC, BC@MnO_2_, and BC@ZnO was effective in reducing the uptake of HMs by ryegrass. In addition, the modified adsorbents were found to be more effective in reducing the bioavailability of HMs. Similar findings to this study were reported by others^18,149–153^. The results of this study show that the adsorbents are able to efficiently immobilize HMs in the soil, reduce their bioavailability to the plant and, on the other hand, prevent their translocation to the aerial parts of the plant. As a result, ryegrass is less dangerous for primary consumers. Zhang et al. (2016) reported that high BCF and TF values indicate higher HMs concentrations in soil, higher uptake by plants and higher translocation to aboveground organs, resulting in increased risk to consumers^154^. These findings suggest that ryegrass has the ability to bind HMs in its roots. For this reason, ryegrass is considered as a suitable phyto-stabilizing plant^155^.

According to our results, the BCF and TF values of Pb were lower than those of the other HMs in all treatments, indicating less uptake by the plant roots and less transfer to the aerial parts. This result supports the findings on metal uptake by plants.

## Conclusion

The main purposes of this study were to synthesis modified biochar with MnO_2_ and ZnO nanoparticles and evaluate and compare their efficiency to remediate HMs contaminated soil and reduce their bioavailability, and improve plant growth. Results showed that ZnO and MnO_2_ nanoparticles were successfully loaded onto biochar (BC). The addition of BC, BC@MnO_2_ and BC@ZnO immobilized Pb, Zn, Cd and Ni in soil, reduced their uptake by the ryegrass and improved the dry weight of the ryegrass, but BC@MnO_2_ and BC@ZnO were more effective than unmodified BC in immobilizing HMs in the soil and reduced their uptake by ryegrass. In addition, the efficiency of immobilization and the reduction of HMs uptake by the plant were higher in treatments with 1% adsorbents than with 0.5%. Overall, our results showed that the application of 1% BC@MnO_2_ was better than other treatments in increasing the ryegrass dry weight, improving immobilization and reducing the availability of HMs to ryegrass. The results showed that adsorbents had a stronger affinity for Pb than the other HMs. It can be concluded that each effectiveness of treatment depends not only on its dose and type but also on the type of HMs. Results of present study showed that biochar modified with MnO_2_ and ZnO nanoparticles had more benefit effect on soils remediation compared to pristine biochar, and they can be used as efficient and low-cost amendments to remediate HMs-contaminated soils and improve plant growth. Also, a comparison of the TF in ryegrass showed that it had a good ability to accumulate all studied HMs in its roots; therefore, it is a suitable plant for HMs phytostabilization.

## Methods

### Materials and chemicals

All chemicals, lead nitrate (Pb(NO_3_)_2_), Zinc nitrate (Zn(NO_3_)_2_), Cadmium nitrate (Cd(NO_3_)_2_), Nickel nitrate (Ni(NO_3_)_2_), potassium permanganate (KMnO_4_), Hydrogen Peroxide (H_2_O_2_), Sodium hydroxide (NaOH), nitric acid (HNO_3_), Zinc sulfate heptahydrate (ZnSO_4_.7H_2_O), and potassium hydroxide (KOH) were provided by Merck company (Germany) and used without any further purification.

### Preparation of biochar

Wheat straw, selected as the raw material for preparing biochar and obtained from the animal husbandry station of Shiraz University Faculty of Agriculture. Wheat straw was first washed several times with deionized water to remove impurities, dried at 60°C for 48 h, and then ground. The crushed samples were pyrolyzed at 500°C in an electric furnace with limited oxygen for three hours^130^. The produced biochar (BC) was passed through a 2-mm sieve and stored in plastic containers.

### Preparation of biochar-loaded with MnO_***2***_ nanoparticles

Biochar modified with MnO_2_ nanoparticles prepared according to the Zhang et al. (2020) and Liang et al. (2017) methods. Briefly, 15 g of BC and 3.16 g of KMnO_4_ (the manganese source) were combined with 150 mL of deionized water and stirred at 25 °C for 30 min. While stirring, 40 ml of 30% H_2_O_2_ was added to the mixture dropwise. After adding 1 M HNO_3_ and NaOH to bring the pH of 7.0, the mixture was stirred for another 30 min and allowed to stand at room temperature for three hours. After filtering, the mixture was repeatedly purified with distilled water and dried at 105 °C for 12 h^66,156^.

### Preparation of biochar-loaded with ZnO nanoparticles

Biochar modified with ZnO nanoparticles was synthesized by the precipitation method. 0.15 M KOH and 0.1 M ZnSO_4_.7H_2_O solutions were prepared in distilled water. 100 mL of 0.1 M ZnSO_4_ solution and 1.0 g of BC were well mixed for 10 min. Then 100 mL of KOH solution was added to the mixture while being vigorously stirred continuously to ensure homogeneity. The mixture was then left to stand for 1 h and then filtered using a Whatman filter. Finally to remove the moisture, the filtered nanocomposite (BC@ZnO) was oven dried at 100°C for 30 h^100^.

### Adsorbent characterization

Scanning electron microscopy (SEM) (TESCAN-Vega3, Czechia) with scattered X-ray spectroscopy (EDX) was used to study the morphology and composition of the adsorbent’s elements. The surface functional groups and the crystal structures of metal minerals of the adsorbents before and after HMs adsorption was characterized by Fourier transform infrared spectroscopy (FTIR) (Tensor II, Bruker, Germany) and X-ray diffractometer (XRD) (Rigaku Ultima IV, Japan) in the 2θ range of 20° to 80°, respectively. The specific surface area of the adsorbents was investigated by Brunauer, Emmett,—Teller (BET) (Belsorp mini II, Microtrac Bel Corp, Japan).

### Soil sampling

The studied soil sample was collected from a depth of 0–30 cm from the Shiraz University Faculty of Agriculture, Bajgah, Fars Province, Iran. The collected soil was air-dried, ground, and passed through a 2 mm sieve. Some physicochemical properties of the studied soil such as particle size by hydrometric method^157^, pH using a pH meter in saturated paste^158^, electrical conductivity (EC) in the extraction using an electrical conductivity meter^159^, cation exchange capacity (CEC) by the replacing cations with NaOAc^160^, organic matter (OM) by the Walkley–Black method^161^. DTPA extraction was used to evaluate the available form of iron (Fe), manganese (Mn), zinc (Zn), copper (Cu), lead (Pb), cadmium (Cd) and nickel (Ni),^162^ and their concentration was determined by the Shimadzu AA-670G atomic absorption spectroscopy(Table 3).

**Table 3:** Some physicochemical properties of studied soil.

Sand (%)	31
Silt (%)	54.2
Clay (%)	14.8
Texture	Silt loam
pH	7.35
EC (dS m^-1^)	2.3
OM (%)	0.985
CEC (Cmol^+^ kg^-1^)	25.2
Mn (mg kg^-1^)	2.35
Pb (mg kg^-1^)	0.3
Cd (mg kg^-1^)	0.25
Zn (mg kg^-1^)	0.895
Ni (mg kg^-1^)	0.15
Fe (mg kg^-1^)	11.72
Cu (mg kg^-1^)	2.73

### Soil contamination and incubation

The soil was divided into four equal sections, and 100 mg kg^-1^ of Zn, Cd, Ni, and Pb (as a nitrate source) were added to each section. The contaminated soil was carefully mixed and incubated for one month at 25°C in a greenhouse condition. Soil samples were irrigated with distilled water during the incubation period, and the soil moisture content was kept at field capacity by adding distilled water^163^. To evaluate the effect of biochar and biochar modified with ZnO and MnO_2_ nanoparticles on the immobilization of Pb, Ni, Cd, and Zn and their bioavailability for ryegrass, a completely randomized design with three replications was conducted under greenhouse conditions. The treatments consisted of: HMs polluted soil (Control) (CK), pristine biochar (0.5% (BC-0.5%), 1% (BC-1%)), ZnO NPs-modified biochar (0.5% (BC@ZnO-0.5%), 1% (BC@ZnO-1%)) and MnO_2_ NPs-modified biochar (0.5% (BC@MnO_2_-0.5%), 1% (BC@MnO_2_-1%)). Adsorbents were added to the contaminated soil, and then they were completely mixed with the soil and incubated for two weeks at a 25 °C temperature and the field capacity moisture.

### Pot experiments and HMs measurments in plant

Ryegrass seeds were selected for cultivation and sterilized in a 1% (*V/V)* NaOCl solution for five minutes. Then, it was washed several times with deionized water and soaked overnight in deionized water. The intubated soils with adsorbents were transferred to pots and 100 perennial ryegrass seeds were planted in each pot^138^.The greenhouse temperature was set steady during the day and night at 25 ± 5 °C. Soil moisture was kept at field capacity by adding distilled water during the growth period (without any leaching). After 7 weeks aerial parts of the plant were harvested and the roots were separated from the soils. To remove any dust particles, the collected shoots were first properly rinsed with tap water and then with distilled water. the roots were washed with tap water, EDTA, and distilled water. A dilute EDTA solution was used to remove HMs that may be present on the surface of the roots. The aerial parts and roots were completely dried at 60 °C for 72 h. After weighting, the dried samples were ground. The concentrations of Cd, Ni, Pb and Zn in the samples were measured by atomic absorption spectrophotometry (AA-6800, Shimadzu) after wet digestion. Equations 1 and 2 were used to determine the HMs uptake by the root and shoot ^163^:

1 $${\text{Root uptake }}\left( {\mu {\text{g pot}}^{{ - { 1}}} } \right) = {\text{root concentration }}\left( {\mu {\text{g g}}^{{ - { 1}}} } \right) \, \times {\text{ root dry matter }}\left( {{\text{g pot}}^{{ - { 1}}} } \right)$$

2 $${\text{Shoot uptake }}\left( {\mu {\text{g pot}}^{{ - { 1}}} } \right) \, = {\text{ shoot concentration }}\left( {\mu {\text{g g}}^{{ - { 1}}} } \right) \, \times {\text{ shoot dry matter }}\left( {{\text{g pot}}^{{ - { 1}}} } \right)$$

### Bio concentration and translocation factors

The effectiveness of biochar and biochar modified with MnO_2_ and ZnO nanoparticles to adsorb HMs and minimize their absorption by plant roots and shoots was determined by transport factor (TF) and bio-concentration factor (BCF) indexes^18^.

3 $${\text{TF}}=\frac{\mathrm{Concentration\,of\,metal\,in\,shoots }(\mathrm{mg kg}-1)}{\mathrm{Concentration\,of\,metal\,in\,roots }(\mathrm{mg kg}-1)}$$

4 $${\text{BCF}}=\frac{Concentration\,of\,metal\,in\,roots\,and\,shoots (mg kg-1)}{concentration\,of\,metal\,in\,soil (mg kg-1)}$$

### Statistical analysis

Data were analyzed using SAS 9.4 software. Differences between treatments were determined following Duncan’s Multiple Range Test (DMRT), (*P* ≤ 0.05). Probability levels of 1% and 5% (*P* ≤ 0.01 or 0.05) were selected to test the significance of the differences. Figures were drawn using Excel 2018 software.

### Ethical approval

No ethical issues were violated in this study.

### Consent to publish

All the authors gave their consent to publishing this manuscript in your journal.

## Data Availability

All data generated or analyzed during this study is included in this article.
